# What law does not understand about public participation

**DOI:** 10.1016/j.heliyon.2024.e32001

**Published:** 2024-05-27

**Authors:** Otelemate Ibim Dokubo, Maria Alina Radulescu, Lorenzo Squintani

**Affiliations:** Faculty of Law, University of Groningen, Groningen, Netherlands

## Abstract

Public participation plays a vital role in developing and implementing climate policies. However, in practice, it is challenging to organise public participation effectively. In this paper, we compare three main regulatory approaches to public participation, the Aarhus Convention, the Escazú Agreement and the Impact and Benefits Agreements, in light of their ability to implement social sciences insights on effective public participation. By linking the social sciences and law, our analysis shows that the shortcomings of each regime, when individually taken, potentially explain why public participation practices face difficulty fulfilling the envisaged goals.

## 1 Introduction

Public Participation is widely acknowledged to play an important role in environmental decision-making, especially in developing and implementing climate policies and adaptation measures [[1], [2], [3]].The reason is the desire to increase the public acceptability of projects, the need to democratise the decision-making process and assist the public in easily understanding scientific facts and producing advanced decisions [4,5]. Given the expected benefits for environmental decision-making processes, several jurisdictions introduced public and private legal regimes for public participation in energy and environmental matters, such as the Arhus Convention (1998), the Escazu Agreement (2018) and the Impact and Benefits Sharing Agreement (IBSA).

### 1.1 Public and private legal instruments for public participation

The Aarhus Convention, which grew out of Principle 10 of the Rio Declaration and the United Nations Agenda 21 program [6], is widely recognised as the most ambitious legislation on public participation and the most important effort to ensure the public's ability to participate in environmental decision-making processes [[7], [8], [9]]. Public participation is covered under Article 6, setting participatory standards regarding the decision-making of specific activities with potential significative adverse effects on the environment, such as the authorisation procedure for a wind park. Besides, Article 7 sets participatory standards for policies, plans and programmes relating to the environment, and Article 8 focuses on generally binding rules, such as subsidy schemes for photovoltaic panels on households' roofs [10].

Based on the Aarhus Convention, Latin American countries adopted the Escazú Agreement [11], which entered into force in 2021 [12]. Article 7 of the Escazú Agreement covers the subject of public participation in specific decisions and other decision-making procedures, such as land-use planning policies, strategies, plans, programmes, rules and regulations, which have or may have a significant impact on the environment. Although this provision resembles several elements within articles 6–8 of the Aarhus Convention, the two instruments are aimed at different jurisdictions and sets of publics, and, as such, we will show that their provisions differ on aspects relevant to this analysis.

Besides the aforementioned public law-based frameworks for public participation, certain jurisdictions have also adopted private law-based legal frameworks, i.e. frameworks based on the self-agreed binding force of agreements between parties. A known private law-based framework for public participation is the Impact and Benefit Sharing Agreements (IBSAs)^2^ employed in resource development. This type of agreement, which only recently gained the attention of scholars and writers, has been used for over a decade to protect indigenous and Aboriginal peoples’ rights to participate in the development of projects carried out on their lands in Australia and Canada [13]. Scholars have proffered various definitions of IBSAs, but we adopt the definition given by Hummel [14]:

′*an agreement executed between a proponent of a project and one or more First Nation Inuit or Metis communities that are potentially impacted by that project’*.

Legal scholars have shown some of the strengths and shortcomings of these instruments; for example, both public law legal frameworks do not provide specific timeframes for public participation, thus pointing to their strengths i.e., flexibility, but also to their weaknesses i.e., the ambiguous nature [7,15]. Furthermore, scholars mainly focused on whether specific procedures complied with existing regulatory standards [16]. Some scholars provided general commentaries about participatory frameworks [17,18] in light of general legal principles, such as legal certainty and non-discrimination [10]. At the same time, the public participation topic has been widely researched by scholars from the social sciences sphere, who provided insights on a variety of aspects, such as the success and hindering factors of public participation processes and offered frameworks for its evaluation [[19], [20], [21]]. Other social sciences scholars focused on the inclusiveness and values-related aspects of public participation [22,23].

Despite the apparent wealth of studies on public participation stemming from both domains, a systematic analysis of the extent to which the main legal frameworks implement social scientific insights on public participation is still missing. This study attempts to fill this knowledge gap by establishing a novel linkage between the domains of law and social sciences regarding public participation. In this way, the present paper seeks to highlight the shortcomings and strengths of the public and private legal instruments and to determine whether further refinements of the analysed regulatory frameworks are necessary to increase the chance of effective public participation. We, therefore, show that law contributes to the difficulties encountered in practice with the organisation of effective public participation procedures by highlighting the mismatch between social sciences insights and regulatory frameworks on public participation. The paper also seeks to highlight which of the legal instruments, be they public or private, align closest to the 4D insights of public participation. Finally, the paper seeks to highlight the level of participation and influence accorded to the diverse public under the private and public legal regimes.

### 1.2 Methodology

The present paper combines a literature review with the analysis of primary legislative sources in light of the normative standards derived from the literature review.

**Fig. 1:**
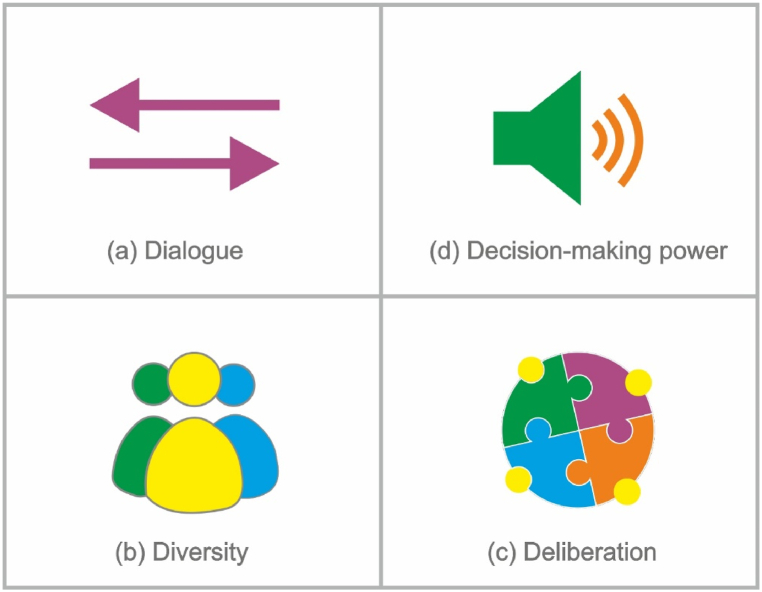
The 4Ds of public participation by Goda Perlaviciute [24]: (a) dialogue; (b) diversity; (c) deliberation; (d) decision-making power.

The study began by sourcing academic articles on public participation and criteria for effective public engagement through an electronic library search. Keywords used during the search include; public participation, successful public participation, public acceptability of energy projects, trust in project developers, benefits of diversity, deliberation in project development, deliberative forums, two-way dialogue, public influence in energy projects, and decision-making power. These articles were systematically reviewed, and an annotated bibliography was compiled to delineate each identified criterion and corresponding arguments. The criteria were then categorised under four main elements: Dialogue, Diversity, Deliberation, and Decision-making Power (4Ds), as shown in Fig. 1.

Additionally, a search was conducted into the most comprehensive legal instruments safeguarding the public's right to participate in the development of energy projects. The Aarhus Convention and the Escazù Agreement emerged as primary legal frameworks and were accessed through the official online repository of the United Nations. Subsequently, a search was conducted to identify private instruments protecting community rights in energy project development, leading to the identification of Impact and Benefit Sharing Agreements.

The provisions within the identified legal instruments pertaining to public participation in project development were identified and grouped according to the established 4D categorisation. To enrich the analysis, supplementary materials such as academic journal articles focusing on public participation provisions, implementation guides, and the Aarhus Convention Compliance Committee Reports were selected. Excerpts from these materials were further categorised under the relevant legal instruments and 4D elements for enhanced comprehension and analysis.

When analysing the factors responsible for the different levels of success of public participation, authors have identified certain concepts that they consider critical for successful public participation procedures, most notably diversity, deliberation, dialogue and decision-making power [[25], [26], [27], [28]]. These criteria have been further synthesized and categorized by Perlavicuite [24], who has described them as the ‘4Ds of public participation’, which we use in this paper as a normative framework (section 1). Employing doctrinal constructivism [29], thus the systematic collection and analysis of primary (laws and jurisprudence) and secondary sources (academic articles and official reports from public bodies), we will assess the extent by which the Arhus Convention (1998), the Escazu Agreement (2018) and the Impact and Benefits Sharing Agreement (IBSA) complies with the 4D theoretical framework (section 3.2). These three legal frameworks for public participation have been chosen because they can be considered the three main legal approaches to public participation. The Aarhus Convention and the Escazú Agreement are both examples of public law-based approaches to public participation. The latter has been inspired by the former and is thus similar in many aspects, but each of them is applicable in different geographical contexts; the Aarhus Convention applies in Europe, and the Escazú Agreement in South America, thus highlighting relevant differences for the analysis presented in this paper. Employing a functional comparative methodology (Michaels, 2006), i.e., the extent by which these different legal instruments and the standards therein prescribed deal with social sciences insights on effective public participation, we will show the strengths and weaknesses of each legal approach (section 2, 3) and discuss the meaning of such findings for the effectiveness of public participation.

## 2 Normative framework: the 4D's of public participation

Fiorino [30] identified three main goals for public participation. Substantively, public participation should aim at better decisions and, thus a higher level of environmental protection. Normatively, public participation should aim at more democratic and legitimised decision-making processes and respect for subjective rights. Functionally, public participation should aim at increasing acceptability. Empirical evidence of public participation procedures that proved effective in achieving these goals is scarce and primarily relates to the functional goal (e.g. in Liu et al., 2019) [31]. Perlaviciute [24] provides a synthesis of the social sciences insights on the criteria for effective public participation. She grouped these insights into four categories of criteria: a) Dialogue, b) Deliberation, c) Diversity, and d) Decision-making power, together called 4D normative standards.

### 2.1 Dialogue

Dialogue, seen as the exchange of information/opinions between at least two parties, is an important criterion for public participation [32,33]. The free flow of information among parties, called two-way dialogue, has been suggested to be crucial to public participation processes [34]. A public participation process involving dialogue leads to a more informed citizenry, increased understanding among parties involved and fosters a relationship among them [26]. ‘Time of engagement’ is an important contributing factor to successful two-way dialogue [35]. Early engagement has been suggested to be critical to the success of public participation processes [36,37].

Furthermore, communication should be complete and transparent. Hall [38] reported how the lack of full communication regarding wind farm projects in the Australian States of New South Wales (NSW) and Victoria in 2009 led to official inquiries from the government. Providing piecemeal information could create perceptions of secrecy within the community, further enhancing the distrust towards the developers. While detailed information is required from developers, it is important to factor in the issue of early communication. Therefore, the appropriate requirement is ‘to engage and communicate as early as detailed information is available’.

We can therefore operationalise the dialogue criterion in light of five standards: a) engagement when all options are still open; b) information about communication method(s), c) sharing of information, d) two-way communication, e) timely and effective communication.

### 2.2 Diversity

In organising public participation processes, it is pertinent that developers acknowledge diversity in terms of age, race, beliefs, values, sex, and education, even within a community. Bringing diverse groups together not only builds community credibility for the developers but also enhances the ‘collective creativity’ and thus leads to more creative and innovative approaches and solutions for the issues at stake [28,33,39]. Yet, adequately representing the population is not easy [40]. The adverse effect is reflected in the limited range of human values captured and addressed in the process [41]. It is, therefore, crucial that all stakeholders are represented as the deliberative process must consider diverse values to lead to good decisions [42].

To enable this, regulatory frameworks should actively include diverse groups based on race, ethnicity, gender, nationality, religion, and language. Furthermore, extra protection and assistance need to be provided to vulnerable groups within the community to enable them to participate. In addition, the right to participate should also be given to both residents and non-residents as long as they are affected by the envisaged activity [43].

### 2.3 Deliberation

While it is imperative for a participatory process to represent the diverse social groups and values of a community [44], diversity could lead to conflicts and distrust among participants, thus causing the process to fall apart or perform worse than one involving homogenous groups [45]. Therefore, the process's outcome depends on how the diverse values are highlighted, aligned and managed by the facilitators of the process [46]. A properly implemented deliberation [27] would allow developers to recognise and address the participants' grievances, provide a range of alternatives and legitimize the decision taken in respect of the project, which would make it easier to implement the decisions taken and increase compliance with same. To improve the efficacy of deliberative forums in achieving their potential benefits, scholars have suggested two dialogic discourses: sense-making discourse and problem-solving discourse, which should alternate at each stage of the process [34]. They state that the sense-making discourse gives room for understanding differences and similarities while the problem-solving discussion leads to an agreement on the project.

In evaluating deliberative methods, Abelson and others [26] have highlighted certain factors necessary for a successful deliberative process. A perusal of the evaluation would reveal one main factor, namely the ‘inclusiveness of the process’ [4]. Factors such as powerplay, knowledge and language differences, communication issues, limited time and opportunity to speak up come into play [26] and can lead to exclusion within the participatory process [40]. The process should allow not only groups who can communicate in a particular way or possess specific knowledge on the subject matter to partake in the process actively [4]. Developers should employ diverse communication methods, such as videos and native interpreters [47]. Furthermore, deliberative forums should adopt techniques such as gaming exercises and situation mapping, allowing everyone to freely share their preferences [34]. Deliberative forums allow developers to gain insights and alternative solutions from persons with traditional knowledge about the environment and energy resources within the area [48].

In light of the above, laws must require the facilitators or relevant persons to ensure that the following factors are considered and addressed within the process: a) power asymmetries, b) rules and guides for the deliberation process, c) sufficient timeframes for parties to present and discuss their preferences, and d) language and communication suited for all process’ participants.

### 2.4 Decision-making power

Deliberative democrats have consistently emphasised the need for extended dialogues and deliberation as an opportunity for debates, the presentation of preferences, and the development of an informed citizenry [49]. Nevertheless, they have also recognised the need to shift from the sense-making discourse to the problem-solving discourse [48]. This view aligns with two of the rungs of Arnstein's [50] ‘ladder of citizen's participation’ known as delegated power and partnership. Here, it is suggested that deliberative processes can confer more decision-making power on the citizens, thereby allowing them to attain a more dominant role in the decision-making process [50].

Scholars [51] suggest that participants would be more likely to be actively interested in the process if they believed they could influence the outcome. If the contrary occurs, participation can be seen as fake and hinder public acceptability of the process and/or project [24]. In this regard [52], showed that project-level decision-making power is affected by what has been decided at the policy and planning level (macro-options). The room for discussing macro-options in general and at the project level thus affects decision-making power.

From a regulatory perspective, the above needs to be translated into a) requirements on the publication of the final decision and of how inputs have been integrated, b) by being taken into account, which means that responsible parties still decide (partial influence) or c) by shaping the outcome of the decision-making process (significant influence), and d) whether decision-making at the macro level is covered as well.

## 3 Findings: Comparison of the alignment of the evaluation of the public and private legal instruments in light of the 4d normative framework

In this section, we present the result of the evaluation of the three legal instruments in light of the 4 Ds (Dialogue, deliberation, diversity and decision-making power).

### 3.1 Dialogue

As shown in Table 1, the Aarhus Convention contains provisions which require all the ingredients for effective dialogue. It recognises the need for two-way communication between both parties, which can be described as dialogue. As prescribed by the Convention, this communication process is divided into active and passive dialogue [16]. Article 6(2) of the Aarhus Convention sets the tone for what can be described as active dialogue. Here, it provides that the concerned public shall be informed, either by a public notice or individually, early in an environmental decision-making procedure and in an adequate, timely and effective manner. This provision further sets the requirement for providing very detailed information about the projects and the identification of the mode of response to the information received and the time allocated for the public to transmit their comments. Thus, the Aarhus Convention provides for communication from the developers, and a means of reciprocal communication from the citizens (Art 6 (2)v). The benefits of this cannot be overstated, as the dialogue usually marks the beginning of the public participatory process. At this stage, community perceptions about the projects typically form. A sense of transparency built through sharing and making available all relevant information, accountability created through feedback and comments, and inclusiveness in formulating options would likely be developed if the Convention is well implemented.

**Table 1:** Below provides a schematic overview of which key criteria for regulating effective dialogue are present under each legal instrument.

Legal Instrument	Contact when all options are still open	Information about communication method(s)	Sharing of project information	Two-way communication	Timely and effective communication
Aarhus Convention	Present	Present	Present	Present	Present
Escazú Agreement	Not Present	Present	Present	Not Present	Present
IBSA	Not Present	Not Present	Present	Present	Present

The Escazú Agreement provides for some of the requirements. Here, the agreement sets it off with the principle of maximum disclosure, which each party must be guided by in implementing the agreement.^3^ This principle is further elaborated with the duty to ensure that the members of the public are equipped with the necessary information in a clear, timely and comprehensive manner,^4^ and through appropriate means which may be in the form of writing, electronic means, orally and by customary means.^5^ This provision already sets it apart from the Aarhus Convention by the inclusion of customary means of communication with the public, and thus recognising the social, economic and cultural differences within the society, which would allow even the most illiterate section of the public to receive and understand the information passed [53]. Nevertheless, unlike the Aarhus Convention, the agreement fails to mandate the states to ensure that participation takes place when all options are still open as required. Furthermore, as regards two-way communication, the Escazú Agreement failed to set specific requirements for the relevant authorities to give instructions on how members of the public can submit their comments in response to the information they receive. As pointed out above, two-way communication is at the core of dialogic processes, as without it, the process is reduced to the mere passing of information. Community members need to believe that they will be given a seat at the table, and this will only happen when they can be involved in the basic step of submitting comments, questions and opinions of the project based on the information they have received. A feeling of non-inclusiveness at the dialogue stage can strain the relationship between the community and the project proponents, negatively affecting the rest of the participatory processes.

The IBSAs recognise the need for two-way communication in a timely manner. The pre-negotiation stage marks the commencement of the process, with project proponents moving to build a collaborative relationship with the community. In this stage, the project's developers engage the community as a whole and distribute information about the project and the company [54]. The information gathered allows the community members to have internal meetings to assess the projects, their capacity constraints, and the level of support they can give the project. The process also involves the negotiation stage requiring both parties to have double-way communication, thus constituting dialogue. While Fidler and Hitch [55] have suggested that the time of engagement for IBSAs is to be determined by the situation or project at hand, Matiation [56]stated that in some cases, IBSA negotiations may be required by the government before the issuance of regulatory approvals or permits [56]. The suggestion of the latter is fairly supported by the Nunavut Land Claims Agreement, which explicitly sets the precondition that the agreement must be entered into and finalised before the commencement of the project.^6^ However, one lawyer specialising in IBSA negotiations argued that the timing may not be early enough as IBSAs may be better negotiated before Environmental Assessment (EA) permits [55]. However, the IBSA guideline [54], Nunavut Agreement and literature have failed to give insight into the method and mode of communicating the project information to the people and the need to contact the communities when all the options are still open, during the dialogue stage. How information is shared is almost as important as sharing the information itself. Community members must receive and understand the information being transmitted. One way to ensure that is to stipulate the need to share that information in an effective and familiar manner with the community. Furthermore, although the IBSAs present the opportunity for both parties to formulate solutions together on the project, the solutions need to be prepared concerning every area of the project.

### 3.2 Diversity

As shown in Table 2, the Escazú Agreement and the IBSA perform equally by including diverse groups and vulnerable persons in the process. A perusal of the IBA guidebook and other literature reveals two forms of participation within the process. First, the project developers engage the entire community directly and share information about the company and the project. Here, the process allows all members to participate without discrimination or selection [54]. The second form of participation is by representatives of the various groups within the community. Here, the stakeholders’ representatives constantly communicate with the group about the issues raised within the negotiation process, collect their views, and present them to the other parties [54]. Reports from First Nation and Aboriginal People show that good communication and a community consultation mechanism allow for more effective and efficient use of leadership and expertise and can avert the divide and conquer mechanism usually employed by companies [57]. Regarding the protection of the participation rights of vulnerable groups, the Agreement is designed to protect indigenous and Aboriginal peoples, categorised as vulnerable groups usually secluded from influencing the decisions of energy projects carried out on their land.

**Table 2:** Provides a schematic overview of the key criteria for regulating diversity present under the Aarhus Convention, Escazu Agreement and the Impact and Benefit Sharing Agreement (IBSA) regimes.

Legal Instrument	Specification for inclusion of various groups	Positive actions to protect vulnerable groups	Protection of non-residents
Aarhus Convention	Not present	Not Present	Present
Escazú Agreement	Present	Present	Not Present
IBSA	Present	Present	Not Present

At the centre of the Escazu Agreement stand its guiding principles enunciated under Article 3, one being the principle of equality and non-discrimination.^7^ It is commendable that a perusal of the entire instrument would reveal these principles represented at various stages of the participatory process. This protection is further enunciated in Art 7(10), which states: ‘*Each Party shall establish conditions that are favourable to public participation in environmental decision-making processes and that are adapted to the social, economic, cultural, geographical and gender characteristics of the public’.* This provision does not just allow for diverse sets of persons within the community to participate in the process but also ensures that they can effectively participate by requiring that favourable conditions are set up, tailored to the social, economic, cultural, geographical and gender characteristics of the public. Furthermore, the Agreement defines vulnerable persons as ‘*persons or groups that face particular difficulties in fully exercising the access rights recognized in the present Agreement, because of circumstances or conditions identified within each Party's national context and in accordance with its international obligations'*.^8^ This definition does not identify persons who can fall under this category but bases the test for vulnerability on difficulty in exercising their rights due to certain restrictions imposed which affect them. While the Aarhus Convention only stipulates provisions for the protection of nationals and non-nationals interested in the project, the Escazú Agreement goes further to place a mandatory obligation on public authorities to *‘identify and* support *persons or groups in vulnerable situations in order to engage them in an active, timely and effective manner in participation mechanisms.* However, it does not regulate non-resident protection, while the Aarhus Convention does [18].

The Aarhus Convention fails to recognise and protect the diverse and vulnerable groups in the community. Nevertheless, in Article 3(9), the Convention sets out a non-discriminatory clause which protects the public's right, both residents and non-residents, nationals and non-nationals, to participate in the process. Reaffirming non-discrimination is important, but it is not the same as actively pursuing equal opportunities, as done by the Escazú Agreement. A one-size-fits-all approach is still possible under the non-discrimination-based approach of the Aarhus Convention, regardless of whether, in practice, certain societal groups cannot make effective use of such a procedure.

### 3.3 Deliberation

The act of deliberation can be inferred from the provisions of Article 6(5) and (7) of the Aarhus Convention, which provide for communication through writing, discussions, comments, analyses, and opinions within public hearings or inquiries. Nevertheless, the Aarhus Convention shows significant shortcomings in ensuring effective deliberation as it only provides for sufficient timeframes for the process under Article 6(3) of the Convention. All other aspects are unregulated. The Escazú Agreement sets out provisions requiring sufficient timeframes,^9^ suitable language of communication^10^ and provisions to deal with power asymmetries.^11^ However, just like the Aarhus Convention, it also fails to require rules or guides for the deliberation process.

On the other hand, the IBSA advances all the essential ingredients, thus recognising the importance of deliberation's success to the entire process. The second stage of the process, known as the negotiation stage, allows the parties (i.e., the companies and elected representatives of all community stakeholders) to present their views, requirements, and preferences about the project [54]. The activities carried out in this stage make it synonymous with the deliberation process. Before the commencement of the negotiation, the parties first enter into a Memorandum of Understanding (MOU), which in no way constitutes consent or acceptance of the project but sets the ground rules for how the negotiations will proceed. The MOU, amongst other things, identifies the representatives on both sides, sets the timetable and budget for the consultation and legal services, and the communications method and channels [57,58]. In support of this process, Sosa and Keenan [57] have argued that although the MOU process may be time-consuming, it sets the rules of the game and, thus, averts future problems such as failure of any party to comply with the obligation during the negotiation, the use of the divide and conquer strategy by companies and leader co-optation. To address the issue of power imbalance, the MOUs also contain the requirement for financial payments to the community to enable them to fund the community's assessment of the project [54]. In addition, the government also plays an important role in levelling the playing field by providing funding, legal protection, training and information to the Aboriginal people [57]. Rules of the game, when properly drafted and implemented, allow parties to avoid potential conflicts arising when parties share their preferences and grievances. It also places certain obligations on all parties to act in a manner that will not jeopardise the process while dealing with power asymmetries and giving silent voices a chance to speak. (See Table 3).

**Table 3:** Provides a schematic overview of which key criteria for regulating effective deliberation are present under the Aarhus Convention, Escazu Agreement and the Impact and Benefit Sharing Agreement (IBSA) regimes.

Legal Instrument	Method/language of communication	Sufficient timeframe	Rules/Guide for deliberation	Dealing with power asymmetries
Aarhus Convention	Not Present	Present	Not Present	Not Present
Escazú Agreement	Present	Present	Not Present	Present
IBSA	Present	Present	Present	Present

### 3.4 Decision- making power

All three instruments promote the communication of the decisions to the community's members through various methods, thus strengthening accountability and transparency even at the end of the participatory process.^12^ The evaluation shows further that the Aarhus Convention and the Escazú Agreement offer partial influence in that the ultimate decision to accept the choices of the public is solely in the hands of the relevant authority which merely has to *take into consideration* the participants' opinions.^13^ Nevertheless, the requirement to take due account of the outcome of public participation does not meet the highest level of participation in Arnstein's ladder of participation, particularly in projects or specific activities.

On the other hand, the IBSA presents an opportunity for the communities to have a seat at the table and decide on what preferences and obligations are accepted and binding on both parties. IBSAs have been described as the best and possibly last option for community members to influence the development of a project that may be inevitable; this is because IBSAs present a different option from the instances where companies merely consult communities to get their opinion on the company's existing plan without the community being able to present a plan of their own [59,60]. Furthermore, O'Faircheallaigh [61] has stated that IBSAs are a means for communities to give their Free Prior and Informed Consent on a project while others [62] have argued that it has created relationships between communities and companies and allowed communities to influence and benefit from project developments in a way that has never been achieved through regulatory processes. While IBSAs are not obligatory and do not override the power of the government to approve projects in a situation where community consent is withheld, it is pertinent to note that it is a binding legal contract [57] and, as such, all parties who enter into the agreement are bound by the terms therein. Therefore, the demands made and agreed to by the people on various issues, including environmental impact mitigation and benefits, are binding on the companies, and can be enforced in case of any breach. In light of the preceding, this paper argues that the level of influence afforded by the IBSA reaches the high levels of Arnstein's ladder of participation (delegated power) by allowing communities to have significant influence over the project development and the opportunity to hold project proponents accountable and eliminate future manoeuvres. This level of influence will not only increase the interest of the people to participate effectively but also create a sense of shared responsibility towards the project's success, thus, leading to more innovative solutions and understanding between the parties.

All three instruments also require the decisions to be timeously publicised or communicated to the public.^14^ Considering the possibility that members of the public may not be satisfied with the decision taken or the extent to which their comments were received or considered, Art 7(9) of the Escazú Agreement further requires that the publicised information on the decision must include the established procedure which would allow the public to take the relevant administrative and judicial actions. Their right to judicial action would also be protected if the requirement to publicise the decision promptly is followed, as the aggrieved persons will have sufficient time to adequately prepare for the matter and meet any time limit for appeal [63]. In addition, Art 6 (9) imposes a further obligation on the decision-makers to promptly inform the public of the decision taken and make accessible the text of the decision together with the reasons and considerations upon which the decision is based. This requirement will expose situations where the public's opinion was ignored or considered and could, in turn, weigh on the decision-makers who need the social license of the public to take into account the public's opinion. More so, public authorities should adopt the Maastricht Recommendation, which states that the statement of reasons accompanying the decision should include a discussion of how the public participation was organized and its outcomes considered and that the statement of reason should be published together with the decision taken.^15^

Furthermore, the Escazu Agreement goes beyond the Aarhus by stipulating methods for dissemination of the decisions to include electronic, oral, and customary methods. Therefore, the issue of certain members of the public not being able to understand the decision taken would be tackled if the relevant authorities apply this provision effectively.

At the same time, the IBSA only focuses on the project level without encompassing the development of policy visions and related plans and programmes. This means that the instrument does not cover macro-options, such as the climate targets or through which energy mix to achieve them. By contrast, the Aarhus Convention and the Escazú Agreement also regulate participatory rights as regards macro by requiring public participation in the preparation of environmental plans and programmes.^16^ On this aspect, they thus score better than IBSA. However, in this case, they also do not allow people to discuss macro-options during participation at the project level [5]. Once again, none of the three investigated instruments fully comply with social sciences insights. (See Table 4)

**Table 4:** Provides a schematic overview of the key criteria for regulating effective decision-making power present under the Aarhus Convention, Escazu Agreement, and the Impact and Benefit Sharing Agreement (IBSA) regimes.

Legal Instrument	Publication/communication of decisions	Partial influence on projects	Significant influence on projects	Coverage of macro-options
Arhus Convention	Present	Present	Not Present	Present
Escazú agreement	Present	Present	Not Present	Present
IBSA	Present	Not Present	Present	Not Present

## 4 Discussion: Towards an integrated regime

The result of this evaluation reveals that all three instruments have their strengths and weaknesses in promoting the 4Ds. While IBSAs present more opportunities for effective deliberation, protection of diverse and vulnerable groups, and real influence over the projects, the Aarhus Convention and Escazú Agreement cope better with dialogue and influence on macro-options at the macro level. The provisions of the Aarhus Convention on the public's right to dialogue have also been reiterated and further elaborated by the Aarhus Convention Compliance Committee (ACCC) in a communication against the Lithuanian authority. Here, it was alleged that the announcement of public participation possibilities in a government publication not normally read by the public, as opposed to the popular daily local newspaper, was ineffective. The Committee held that the public should be informed in a manner that represents a reasonable chance to participate and that information in a weekly journal does not satisfy the requirement under the law.^17^

This requirement of the Convention for early and timely dissemination of project information has also been enforced by the Aarhus Convention Compliance Committee (ACCC) in a communication submitted by an Austrian-based NGO, Global 2000/Friends of the Earth, against the Slovakian government in 2009, alleging a breach of Article 6. The Committee held that the government was in breach of Article 6 (4) and (10) of the Convention because they failed to provide for early and effective public participation in the decision-making process leading to the decision regarding the Mochovce Nuclear Power Plant.^18^ It is also interesting to notice that the two public law-based frameworks analysed differ in how they translate social sciences insights into regulatory standards. Most notably, as regards diversity, the two regimes differ. The 2 frameworks were drafted to fit the characteristics of the communities they were enacted to regulate. In Europe, the presence of indigenous people is different than in Latin America, thus explaining the different approaches followed under the two regimes. Nevertheless, certain characteristics such as gender and class are present within every society and should be considered while preparing regulatory instruments. It is therefore arguable that also under the Aarhus Convention, positive actions to support the participation of vulnerable societal groups should be fostered [64].

Also, the difference concerning the protection of non-residents under the Aarhus Convention and the Escazú Agreement could be linked to the different geopolitical developments of the interested regions. Indeed, the drafting of the Aarhus Convention saw the participation of the European Union as a Party to the Convention. The provision of the Convention has also been reiterated and applied by the Aarhus Convention Compliance Committee (ACCC) in an advice to the Kazakhstan government, where the Committee stated that *‘special arrangements may need to be put in place during the pandemic to ensure that the foreign public can participate in that decision-making process without discrimination as to citizenship, nationality or domicile’*.^19^ Non-discrimination based on residence or nationality among the Member States is proactively countered within the European Union.

As for the criterion of dialogue, only the Aarhus Convention is in full compliance with the social sciences' insights on effective public participation. The same applies to the decision-making power criterion, regarding the possibility of discussing macro-options at the project level as the IBSA fails to provide for macro-options at the project level [5]. It is disarming to notice that none of the analysed frameworks is aligned with the social sciences’ insights on effective public participation. This explains why public participation does not seem to deliver on its goals in practice. Indeed, even if the regulatory standards established under each regulatory regime, individually taken, were complied with, the participatory practice will still fall short on one or more criteria for effective public participation. Responsible parties could decide on a case-by-case basis to integrate the legal requirements regarding the missing criteria, but this would require an active search for such criteria in scientific literature, and there is no system for the oversight of whether this occurs and, if it occurs, how.

Despite the individual shortcomings, it is encouraging that a combined reading of the various regimes does fulfil most, although not all, the criteria stemming from social sciences insights. The best framework would be one which integrates the strengths of all three instruments, as none of the legal instruments is solely capable of fulfilling all the ingredients of the 4D theory. First, public law-based frameworks modelled on those from a combination of the provisions from the Aarhus Convention and Escazu Agreement can set the basis for dialogue and decision-making power throughout the whole decision-making chain. Secondly, the standards from the public law-based framework can be integrated using private law instruments modelled on the IBSAs, allowing for refinement of all aspects of public participation with particular attention to the regulation of deliberative processes at a project level.

## 5 Conclusion

The journey towards achieving effective public participation in energy projects requires the collective efforts of the government and its institutions, the energy companies, and all relevant stakeholders. In this case, the role of the government lies in the creation and implementation of public participation policies to be applied within the energy and environmental sector. It is because of this obligation that laws such as the Aarhus Convention and the Escazu Agreement have been enacted and adopted by various states. Nevertheless, private legal instruments such as the IBSAs have also been adopted by certain jurisdictions to address the issue of inadequate public participation of communities in projects that affect them. This paper therefore found it imperative that both the private and public legal instruments should be evaluated through the lens of a normative standard (in this case, the 4d theory). The findings of this paper revealed that all three instruments have their strengths and shortcomings in promoting the 4Ds. This paper acknowledges the advancement of the IBSAs in achieving most of the ingredients necessary for successful public participation, however, the best framework would be one which integrates the strengths of all three instruments as none is solely capable of fulfilling all the ingredients of the 4D theory.

### 5.1 Limitations of the study

Desk research did not pose challenges in terms of data access, but it also may not provide enough depth or insight into qualitative aspects. However, this is the first article in a series of articles that the main author is working on, and the upcoming articles will also bring empirical data in order to bring qualitative aspects in relation to the 4Ds. This is also a starting point for other researchers to address the inadequacies of regulatory frameworks in fulfilling insights for effective public participation.

Furthermore, this paper does not include an analysis of national implementations of the 3 frameworks. A more comprehensive analysis could show that the minimalistic approach at the level of international public law is integrated with additional standards at the national level, improving the picture. In reality, national laws do not seem to go beyond international standards, but this has not been assessed in this paper.

## Data availability

Has data associated with your study been deposited into a publicly available repository?

No, because the research was purely desk research and data used in this paper was obtained from academic journals, websites, and regulatory papers.

No data from empirical research was used for the research described in the article.

## CRediT authorship contribution statement

**Otelemate Ibim Dokubo:** Writing – original draft, Resources, Methodology, Formal analysis, Conceptualization. **Maria Alina Radulescu:** Writing – review & editing, Supervision. **Lorenzo Squintani:** Writing – review & editing, Supervision, Conceptualization.

## Declaration of competing interest

The authors declare that they have no known competing financial interests or personal relationships that could have appeared to influence the work reported in this paper.

## Footnotes

2 They are also known as Benefit Agreements, Impact and Benefit Agreements or participation agreements (Indigenous Bar Association, 2004, p. 20).

3 Art 3(h) of the Escazu Agreement.

4 Art 7 (4) of the Escazu Agreement.

5 Art 7 (6) of the Escazu Agreement.

6 See section 26 (2) 1) of the Agreement < available at < https://nlca.tunngavik.com/?page_id=2444#ANCHOR2445 > accessed on the 8th of May 2023.

7 Art 3 of the Escazu Agreement states as follows; Each Party shall be guided by the following principles in implementing the present Agreement: (a) Principle of equality and principle of non-discrimination; (b) Principle of transparency and principle of accountability; (c) Principle of non-regression and principle of progressive realization; (d) Principle of good faith; (e) Preventive principle; (f) Precautionary principle; (g) Principle of intergenerational equity; (h) Principle of maximum disclosure; (i) Principle of permanent sovereignty of States over their natural resources; (j) Principle of sovereign equality of States; and (k) Principle of pro persona.

8 See Art 2 (e) of the Escazu Agreement.

9 Art 7(5) of the Escazu Agreement.

10 Art 7(11) of the Escazu Agreement.

11 Article 7(10) of the Escazu Agreement.

12 See Article 6(9) of the Aarhus Convention; Article 7(8) and (9) of the Escazu Agreement; the IBSA Guidebook by Ref. [54].

13 Article 6(8) of the Aarhus Convention; Article 7(7) of the Escazu Agreement.

14 See 6(9) of the Aarhus Convention, Art 7(8) of the Escazu Agreement and the IBA Guidebook e.

15 See Para 130 and 131 of the Maastricht Recommendations on Promoting Effective Public Participation in Decision-making in Environmental Matters.

16 See Article 7(3) of the Escazu Agreement and Article 7 of the Aarhus Convention.

17 U.N. Econ. Comm'n for Eur., Report by the Compliance Committee on its third meeting: Compliance by Lithuania with its Obligations Under the Convention, 16, U.N. Doc. ECE/MP. PP/2008/5/Add.6 (Apr. 4, 2008.

18 U.N. Econ. Comm'n for Eur., Report by the Compliance Committee on its fourth meeting: Compliance by Slovakia with its Obligations Under the Convention, 36, U.N. Doc. ECE/MP.PP/2011/11/Add.3 (December.13,2010.

19 Recommendations with regard to a request for advice ACCC/A/2020/2 by Kazakhstan; ACCC/A/2020/2 (Kazakhstan) (ECE/MP.PP/C.1/2021/6).

## References

[1] Uittenbroek C.J., Mees H.L.P., Hegger D.L.T., Driessen P.P.J. (2019). The design of public participation: who participates, when and how? Insights in climate adaptation planning from The Netherlands. J. Environ. Plan. Manag..

[2] Intergovernmental Panel on Climate Change (IPCC) (2023).

[3] Cattino M., Reckien D. (2021). Does public participation lead to more ambitious and transformative local climate change planning?. Curr. Opin. Environ. Sustain..

[4] Barnes M. (1999). Researching public participation. Local Gov. Stud..

[5] Perlaviciute G., Squintani L. (2020). Public participation in climate policy making: toward reconciling public preferences and legal frameworks. One Earth.

[6] Petts J., Leach B. (2000).

[7] Lee M., Abbot C. (2003). The usual suspects? Public participation under the Aarhus convention. Mod. Law Rev..

[8] Palerm J.R. (1999). Public participation in environmental decision making: examining the Aarhus convention. J. Environ. Assess. Policy Manag..

[9] Barritt E. (2020).

[10] Squintani L.1981-, Darpö J.1952-, Lavrysen L., Stoll P.-T. (2019).

[11] Dávila A.S. (2022).

[12] Pánovics A. (2021).

[13] O'Faircheallaigh, Aborigines C. (2006). Mining companies and the state in contemporary Australia: a New political economy or ‘Business as usual’. Aust. J. Polit. Sci..

[14] Hummel C. (2019). Behind the Curtain, impact benefit agreement transparency in Nunavut. Cah. Droit.

[15] Zhao Y., Butcher B. (2022). Coming to terms with public participation in decision making: balancing clarity and impact in the Aarhus convention. Rev. Eur. Comp. Int. Environ. Law.

[16] Jendroska J. (2005). Aarhus convention and community law: the interplay. J. Eur. Environ. Plan. Law.

[17] Ebbesson J., Gaugitsch H., Jendroska J., Marshall F., Stec S. (2014).

[18] Stec S., Jendrośka J. (2019). The Escazú agreement and the regional approach to Rio principle 10: process, innovation, and shortcomings. J. Environ. Law.

[19] Liu B., Hu Y., Wang A., Yu Z., Yu J., Wu X. (2018). Critical factors of effective public participation in sustainable energy projects. J. Manag. Eng..

[20] Reed M.S., Vella S., Challies E., De Vente J., Frewer L., Hohenwallner‐Ries D., Huber T., Neumann R.K., Oughton E.A., Sidoli del Ceno J. (2018). A theory of participation: what makes stakeholder and public engagement in environmental management work?. Restor. Ecol..

[21] Rowe G., Frewer L.J. (2000). Public participation methods: a framework for evaluation. Sci. Technol. Hum. Values.

[22] Brown G., Reed P., Raymond C.M. (2020). Mapping place values: 10 lessons from two decades of public participation GIS empirical research. Appl. Geogr..

[23] Perlaviciute G., Squintani L. (2023). Time to talk about values, time to say No: what drives public participation in decision-making on abstract versus concrete energy projects?. PLOS Clim.

[24] Perlaviciute G. (2021). Contested climate policies and the four ds of public participation: from normative standards to what people want. WIREs Clim. Change.

[25] Dryzek J.S. (1990).

[26] Abelson J., Forest P.-G., Eyles J., Smith P., Martin E., Gauvin F.-P. (2003). Deliberations about deliberative methods: issues in the design and evaluation of public participation processes. Soc. Sci. Med..

[27] Blacksher E., Diebel A., Forest P.-G., Goold S.D., Abelson J. (2012). What is public deliberation. Hastings Cent Rep.

[28] Ellemers N., Rink F. (2016). Diversity in work groups. Curr. Opin. Psychol..

[29] Von Bogdandy A. (2010). Founding principles of EU law: a theoretical and doctrinal Sketch. Eur. Law J..

[30] Fiorino D.J. (1990). Citizen participation and environmental risk: a survey of institutional mechanisms. Sci. Technol. Hum. Values.

[31] Liu L., Bouman T., Perlaviciute G., Steg L. (2019). Effects of trust and public participation on acceptability of renewable energy projects in The Netherlands and China. Energy Res. Soc. Sci..

[32] Swidler L., Swidler L. (2014).

[33] Innes J.E., Booher D.E. (2004). Reframing public participation: strategies for the 21st century. Plan. Theory Pract..

[34] Hamilton J.D., Wills-Toker C. (2006). Reconceptualizing dialogue in environmental public participation. Policy Stud. J..

[35] Hanna P., Vanclay F., Langdon E.J., Arts J. (2014). Improving the effectiveness of impact assessment pertaining to indigenous peoples in the Brazilian environmental licensing procedure. Environ. Impact Assess. Rev..

[36] Hindmarsh R. (2010). Wind farms and community engagement in Australia: a critical analysis for policy learning. East Asian Sci. Technol. Soc. Int. J..

[37] Anderson R.A., Plowman D., Corazzini K., Hsieh P.-C., Su H.F., Landerman L.R., McDaniel R.R. (2013). Participation in decision making as a property of complex adaptive systems: developing and testing a measure. Nurs. Res. Pract..

[38] Hall N.L. (2014). Can the “social licence to operate” concept enhance engagement and increase acceptance of renewable energy? A case study of wind farms in Australia. Soc. Epistemol..

[39] Rădulescu M.A., Leendertse W., Arts J., Franklin A. (2022). Co-Creativity and Engaged Scholarship: Transformative Methods in Social Sustainability Research.

[40] Parkins J.R., Mitchell R.E. (2005). Public participation as public debate: a deliberative turn in natural resource management. Soc. Nat. Resour..

[41] Rockloff S.F., Moore S.A. (2006). Assessing representation at different scales of decision making: rethinking local is better. Policy Stud. J..

[42] Dietz T. (2013). Bringing values and deliberation to science communication. Proc. Natl. Acad. Sci..

[43] Dällenbach N., Wüstenhagen R. (2022). How far do noise concerns travel? Exploring how familiarity and justice shape noise expectations and social acceptance of planned wind energy projects. Energy Res. Soc. Sci..

[44] Beierle T.C., Konisky D.M. (2000). Values, conflict, and trust in participatory environmental planning. J. Policy Anal. Manage..

[45] Maznevski J., Di Stefano J. (2000). Creating value with diverse teams in global management. Organ. Dyn..

[46] Rădulescu M.A., Leendertse W., Arts J. (2020). Conditions for Co-creation in infrastructure projects: experiences from the overdiepse polder project (The Netherlands). Sustainability.

[47] Hanna P., Vanclay F., Arts J. (2016). The communication and management of social risks and their relevance to corporate-community relationships. Commun. Risk.

[48] Fischer F. (2000).

[49] Wellstead A.M., Stedman R.C., Parkins J.R. (2003). Understanding the concept of representation within the context of local forest management decision making. For. Policy Econ.

[50] Arnstein S.R. (1969). A ladder of citizen participation. J. Am. Inst. Plann..

[51] McCool S.F., Guthrie K. (2001). Mapping the dimensions of successful public participation in messy natural resources management situations. Soc. Nat. Resour..

[52] Squintani L., Perlaviciute G. (2022).

[53] André P., Enserink B., Conner D., Croal P. (2006).

[54] Gunton C., Batson J., Gunton T., Markey S., Dale D. (2020).

[55] Fidler C., Hitch M. (2007). Impact and benefit agreements: a contentious issue for environmental and Aboriginal Justice. Environments.

[56] Matiation S. (2002). Impact benefits agreements between mining companies and aborginal communities in Canada: a model for natural resource developments affecting indigenous groups in Latin America. Gt. Plains Nat Resour. J.

[57] Sosa I., Keenan K. (2001).

[58] Knotsch C., Warda J. (2009).

[59] Caine K.J., Krogman N. (2010). Powerful or just plain power-full? A power analysis of impact and benefit agreements in Canada's North. Organ. Environ..

[60] St-Laurent G.P., Le Billon P. (2015). Staking Claims and shaking hands: impact and benefit agreements as a technology of government in the mining sector. Extr. Ind. Soc..

[61] O'Faircheallaigh C. (2010). Aboriginal-mining company contractual agreements in Australia and Canada: implications for political autonomy and community development. Can. J. Dev. Stud. Can. Détudes Dév..

[62] Prno J., Bradshaw B., Lapierre D. (2010).

[63] Etemire U. (2023). Public voices and environmental decisions: the Escazú agreement in comparative perspective. Transnatl. Environ. Law.

[64] Squintani L., Schoukens H. (2019).

